# Case Report: Central retinal artery occlusion following sildenafil intake

**DOI:** 10.12688/f1000research.122087.1

**Published:** 2022-06-01

**Authors:** Anis Mahmoud, Fatma Abid, Molka Khairallah, Fatma Sakji, Hassen Ibn Hadj Amor, Hala Attia, Sameh Mbarek, Riadh Messaoud

**Affiliations:** 1 Department of Ophthalmology, Tahar Sfar University Hospital, Mahdia, 5100, Tunisia; 2 Faculty of Medicine, University of Monastir, Monastir, Tunisia; 3 Department of Cardiology, Tahar Sfar University Hospital, Mahdia, 5100, Tunisia; 4 Department of Anesthesiology and Perioperative Medicine, Tahar Sfar Hospital, Mahdia, 5100, Tunisia

**Keywords:** Central retinal artery occlusion, Systemic Drug Retinal Toxicity, Sildenafil, phosphodiesterase V inhibitor.

## Abstract

**Purpose:** To report a case of central retinal artery occlusion associated with sildenafil intake and briefly discuss its causative pathogenesis.

**Methods: **A 50-year-old man with no premorbidities presented with symptoms of sudden severe visual field constriction in the left eye (LE). Best-corrected visual acuity in the LE was 20/25. Fundus examination and fluorescein angiography of the LE were suggestive of central retinal artery occlusion (CRAO) with cilioretinal artery sparing. Further investigation revealed that 100 mg of sildenafil had been taken for the first time three hours before the onset of symptoms.

**Results: **The patient was treated promptly with intravenous acetazolamide, sublingual isosorbide dinitrate and ocular massage, but without visual recovery. No other associated systemic or local risk factors were found, and the case was classified as a potential complication of sildenafil.

**Conclusion: **Although no direct link could be established, the aim of this report is to highlight the incidence and to consider this issue when evaluating any case of central retinal artery occlusion.

## Introduction

Sildenafil is a specific phosphodiesterase V inhibitor which is a widely used treatment for erectile dysfunction. Many reports have highlighted ischemic ocular side effects associated with sildenafil. ^1^ We report herein a case of central retinal artery occlusion (CRAO), which occurred a few hours after oral sildenafil intake.

## Case report

**Figure 1.**
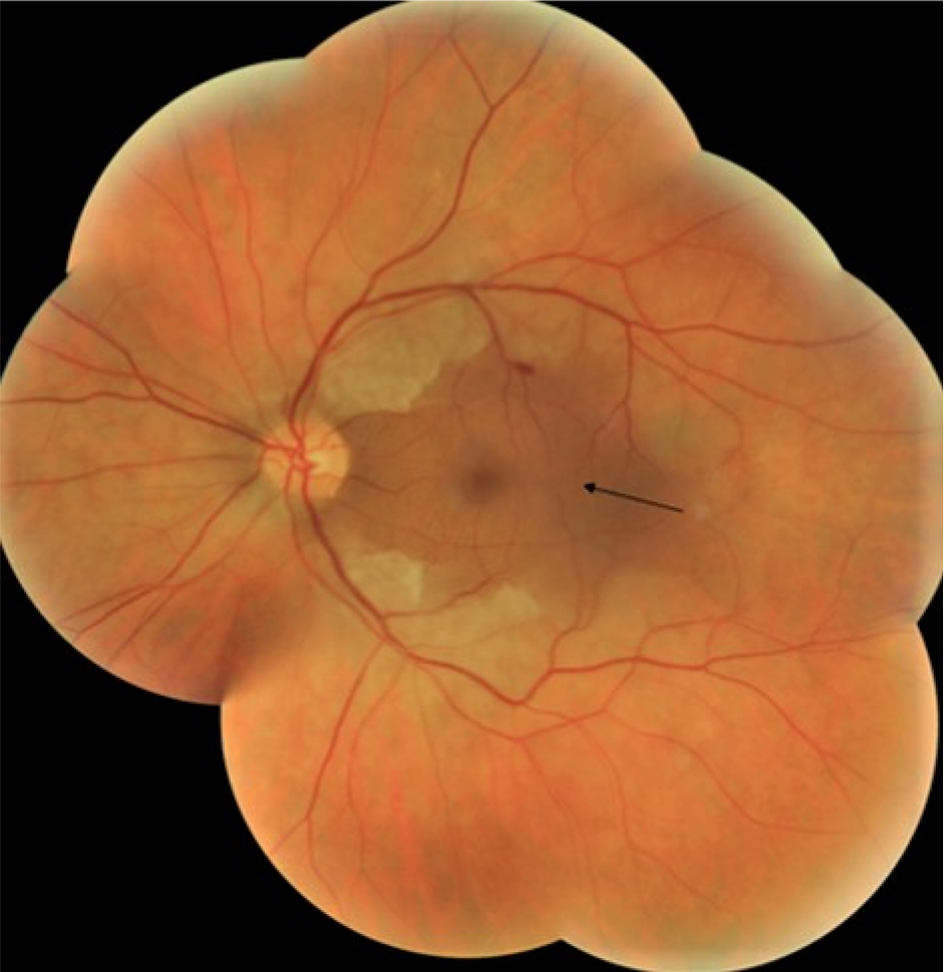
Baseline composite fundus photograph of the left eye showing retinal whitening except for the cilioretinal artery distribution area (black arrow).

A 50-year-old Tunisian man, otherwise healthy and unemployed, presented to the ophthalmology department with sudden severe visual field constriction in the left eye (LE) of 48 hours duration preceded by severe headaches. On ophthalmic examination, visual acuity was 20/20 in the right (RE) eye and 20/25 in the left eye (LE). LE fundus examination revealed diffuse faint retinal whitening, except for central area of normal retinal color along the distribution of a perfused cilioretinal artery ( Figure 1: black arrow).

**Figure 2.**
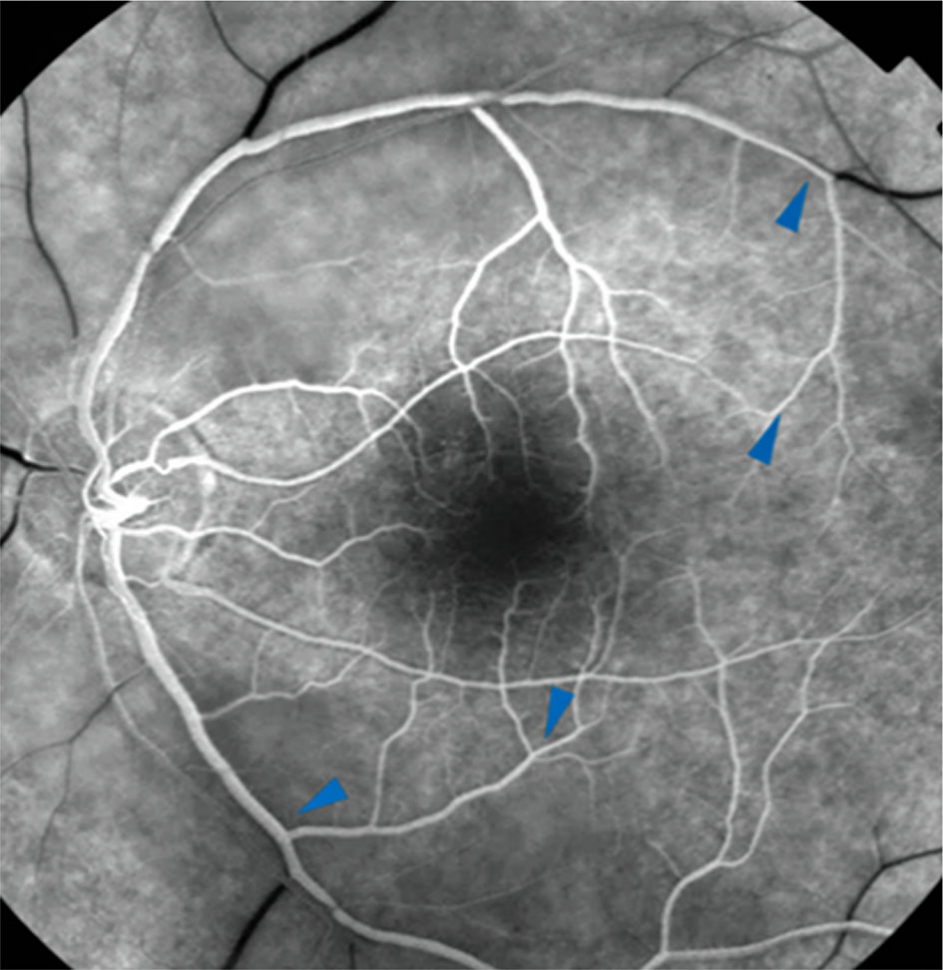
Fluorescein angiography shows interruption of flow in the central retinal artery, regular filling of the cilioretinal artery and late retrograde filling in the central retinal vein.

Anterior segment examination of both eyes as well as fundus examination of theRE were unremarkable. LE fluorescein angiography (FA) showed no filling of the central retinal artery, regular filling of the cilioretinal artery and late retrograde filling of the central retinal vein ( Figure 2: blue arrowheads).

**Figure 3.**
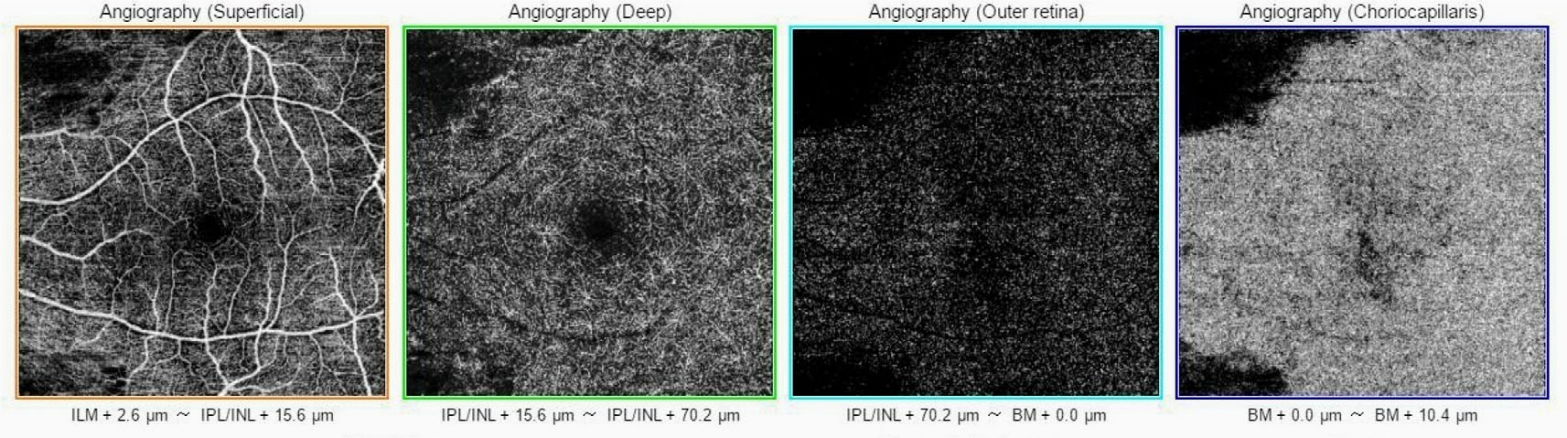
Baseline OCT-A of the left eye shows no flow in superficial and deep retinal capillary plexuses microvasculature. Only the flow in cilioretinal artery is visible.

OCT-A of the left eye at first presentation shows no flow in the microvasculature of the superficial and deep retinal capillary plexuses, except for the territory of the cilioretinal artery ( Figure 3).

LE central retinal artery occlusion (CRAO) with cilioretinal artery sparing was diagnosed. Echocardiogram, carotid artery imaging and blood tests were unremarkable. Further investigation revealed that a 100 mg dose of sildenafil had been taken for the first time three hours before the onset of symptoms.

Ocular massage was performed as well as sublingual isosorbide dinitrate and intravenous acetazolamide were administered. Two weeks later, reduction in retinal oedema was evident on left eye fundus examination, although there was no improvement in either visual acuity or visual field.

## Discussion

Various ischemic ocular events related to sildenafil have been reported. The most notable are branch retinal artery occlusion, ^2^ acute macular neuroretinopathy, ^3^ anterior ischemic optic neuropathy, ^4^^,^^5^ central retinal vein occlusion ^6^ and cilioretinal artery occlusion. ^7^

Only two cases of CRAO with sildenafil have been reported in the literature. ^8^^,^^9^

The patient presented here reported taking sildenafil (100 mg) a few hours before the onset of ocular symptoms and headaches. In fact, ocular side effects are directly proportional to the blood concentration of the drug, which usually appears between 15 and 30 minutes after administration, reaches a peak one to two hours later and clears halfway in 3 to 5 hours. ^10^

As sildenafil has a high systemic vasodilator effect that reduces systemic blood pressure, ^11^ it may decrease cerebral blood flow leading to severe headaches as experienced by our patient. Similarly, numerous clinical studies have demonstrated that sildenafil induces retinal veinous vasodilatation *in vivo.* ^12^

Both the absence of risk factors for retinal vascular occlusion and the timeline of events indicates that oral sildenafil was probably a contributing factor in the development of CRAO, but its pathogenesis remains speculative. We suggest that central retinal artery occlusion occurs in the region of the lamina cribrosa where the central retinal vein and artery share a common adventitia. We speculate that sildenafil-related vasodilation of the central retinal vein causes central retinal artery compression, resulting in secondary changes, including blood flow changes, endothelial damage and platelet thrombi, leading to CRAO. This case underlines the importance of a careful drug intake investigation in cases of CRAO without obvious cause. This incident should not be overlooked by physicians and must be seriously discussed with patients requiring sildenafil, especially since most of them are at risk for ocular ischemic events. Nevertheless, this association remains poorly explained and requires further documented cases.

## Conclusion

In summary, CRAO secondary to sildenafil is extremely rare and has only been reported in the literature twice previously. Clinicians should be aware of this risk and should avoid prescribing sildenafil in patients with a history of ischemic ocular events.

## Data availability

All data underlying the results are available as part of the article and no additional source data are required.

## Consent

Written informed consent for publication of their clinical details and clinical images was obtained from the patient.
