# Supplementary material for: Case Report: Central retinal artery occlusion following sildenafil intake
Source: F1000Res. 2022 Jun 1;11:600. [Version 1] doi: 10.12688/f1000research.122087.1 (PMC9490277; doi:10.12688/f1000research.122087.1)
Supplement: Peer review history

# Peer review: Reviewer response for version 1

**DOI:** 10.5256/f1000research.134029.r150351

**Published:** 2022-09-20

**Authors:** Wassim Ben Hadj Salah

**Affiliations:** 1 Service d’Ophtalmologie, Centre Hospitalier Louis Pasteur, Le Coudray, France

The author reported an original case of a young adult patient with no personal history who presented with a central retinal artery occlusion following the intake of Sildenafil.

 The retinal imaging is interesting, moreover, it would be necessary to add the early times of the retinal angiography and a B mode OCT,  as well as it should be specified that the imaging was not done at an acute stage of the arterial occlusion seen the blood flow recovery exceeding the cilioretinal artery area.

 It may be relevant to detail the biological radiological and clinical explorations done to rule out other etiologies of retinal arterial occlusion like cardiovascular pathologies, dysimmune diseases with vasculitis, and hematological disorders

Are enough details provided of any physical examination and diagnostic tests, treatment given and outcomes?

Yes

Is the case presented with sufficient detail to be useful for other practitioners?

Yes

Is sufficient discussion included of the importance of the findings and their relevance to future understanding of disease processes, diagnosis or treatment?

Yes

Is the background of the case’s history and progression described in sufficient detail?

Yes

Reviewer Expertise:

Ophtalmology Ocular Surface Disease , glaucoma, retinal diseases, anterior segment surgery, contactology

I confirm that I have read this submission and believe that I have an appropriate level of expertise to confirm that it is of an acceptable scientific standard.

# Peer review: Reviewer response for version 1

**DOI:** 10.5256/f1000research.134029.r139570

**Published:** 2022-06-21

**Authors:** Yousra Falfoul

**Affiliations:** 1 Oculogenetic Laboratory LR14SP01, Faculty of Medicine of Tunis, Hedi Rais Institute of Ophthalmology (Department B), Tunis El Manar University, Tunis, Tunisia

The authors report a case of central retinal artery occlusion as a potential complication of Sildenafil. they conclude that Clinicians should take into account this complication while prescribing this treatment. 

 In this case, multimodal imaging was well detailed with high quality images. It would be also interesting if the authors could add structural OCT imaging of the posterior pole. On LE fluorescein angiography, are there more earlier phases that could better describe the retrograde filling of the posterior pole vessels? Did the general examination of the patient reveal some characteristics that could be possible markers predicting this potential complication when prescribing this treatment (obesity, sleep apnea syndrome, recent vaccination, COVID...?)

Are enough details provided of any physical examination and diagnostic tests, treatment given and outcomes?

Yes

Is the case presented with sufficient detail to be useful for other practitioners?

Yes

Is sufficient discussion included of the importance of the findings and their relevance to future understanding of disease processes, diagnosis or treatment?

Yes

Is the background of the case’s history and progression described in sufficient detail?

Yes

Reviewer Expertise:

retinal imaging, peridatric ophthalmology, cataract surgery, oculogenetics

I confirm that I have read this submission and believe that I have an appropriate level of expertise to confirm that it is of an acceptable scientific standard.
